# Phylogeography and sexual macrocyst formation in the social amoeba *Dictyostelium giganteum*

**DOI:** 10.1186/1471-2148-10-17

**Published:** 2010-01-20

**Authors:** Natasha J Mehdiabadi, Marcus R Kronforst, David C Queller, Joan E Strassmann

**Affiliations:** 1 Department of Ecology and Evolutionary Biology, Rice University, Houston, Texas, 77005, USA; 2 Department of Entomology and Laboratories of Analytical Biology, National Museum of Natural History, Smithsonian Institution, P. O. Box 37012, Washington, DC 20013-7012, USA; 3 FAS Center for Systems Biology, Harvard University, 52 Oxford Street, Cambridge, Massachusetts 02138, USA

## Abstract

**Background:**

Microorganisms are ubiquitous, yet we are only beginning to understand their diversity and population structure. Social amoebae (Dictyostelia) are a diverse group of unicellular eukaryotic microbes that display a unique social behaviour upon starvation in which cells congregate and then some die to help others survive and disperse. The genetic relationships among co-occurring cells have a major influence on the evolution of social traits and recent population genetic analysis found extensive genetic variation and possible cryptic speciation in one dictyostelid species (*Dictyostelium purpureum*). To further characterize the interplay among genetic variation, species boundaries, social behaviour, and reproductive isolation in the Dictyostelia, we conducted phylogenetic analyses and mating experiments with the geographically widespread social amoeba *Dictyostelium giganteum*.

**Results:**

We sequenced approximately 4,000 basepairs of the nuclear ribosomal DNA from 24 isolates collected from Texas, Michigan, Massachusetts, Virginia, and Wisconsin and identified 16 unique haplotypes. Analyses of the sequence data revealed very little genetic differentiation among isolates and no clear evidence of phylogenetic structure, although there was evidence for some genetic differentiation between the Massachusetts and Texas populations. These results suggest that sexual mating (macrocyst formation) is not likely to correlate with either genetic or geographical distance. To test this prediction, we performed 108 mating experiments and found no association between mating probability and genetic or geographical distance.

**Conclusions:**

*D. giganteum *isolates from across North America display little genetic variation, phylogeographic structure, and genetic differentiation among populations relative to the cryptic species observed within *D. purpureum*. Furthermore, variation that does exist does not predict the probability of mating among clones. These results have important implications for our understanding of speciation and social evolution in microbes.

## Background

Studies of microbial biogeography and diversity provide a better understanding of the population structure, intraspecific genetic differentiation, and genetic diversity of these ubiquitous organisms [1,2]. Unlike plants and animals, free-living microorganisms are predicted to exhibit little population structure because their small size and large numbers make them easily dispersed [reviewed in [3,4]]. If microbes are characterized by high gene flow, then this should decrease microbial diversity across the landscape [5-7]. However, several studies have found that microorganisms can exhibit biogeographical patterns [e.g., [8-16]]. Distinguishing between these two alternative hypotheses is especially important for social microorganisms because population structure affects social interactions [17].

Social amoebae live in decaying vegetative matter that forms the top layers of soil worldwide [18]. These social microorganisms are in the Amoebozoa, the sister group of fungi plus animals [19,20]. Most of the time, social amoebae exist as single cells that prey upon bacteria. However, when bacteria become scarce, tens of thousands of cells aggregate to form a multicellular fruiting body in which some amoebae die to form a stalk that supports the remaining cells that then differentiate into living drought-hardy spores [21,22]. The stalk holds aloft the spores from hazards of the soil [23] and facilitates transport, and when conditions are favorable, the spores hatch and separate into individual amoebae.

**Figure 1:**
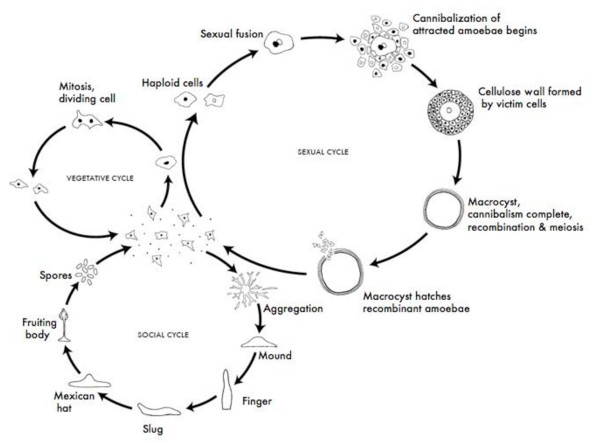
**The life cycles of *Dictyostelium***. Most of its life, this haploid social amoeba undergoes the vegetative cycle, preying upon bacteria in the soil, and periodically dividing mitotically. When food is scarce, either the sexual cycle or the social cycle begins. Under the social cycle, amoebae aggregate to cAMP by the thousands, and form a motile slug, which moves towards light. Ultimately the slug forms a fruiting body in which about 20% of the cells die to lift the remaining cells up to a better place for sporulation and dispersal. Under the sexual cycle, amoebae aggregate to cAMP and sex pheromones, and two cells of opposite mating types fuse, and then begin consuming the other attracted cells. Before they are consumed, some of the prey cells form a cellulose wall around the entire group. When cannibalism is complete, the giant diploid cell is a hardy macrocyst, which eventually undergoes recombination and meiosis, and hatches hundreds of recombinants. Not drawn to scale. *CC Creative Commons Attribution - Share Alike 3.0, David Brown & Joan E. Strassmann*.

Sexual mating (i.e., macrocyst formation) in dictyostelids can occur if the right environmental conditions are met. This alternative life cycle to asexual fruiting body formation happens under high humidity and darkness [18,22] (Figure 1). Haploid cells of the appropriate mating types (i.e., pairing of strains that results in the production of macrocysts) fuse to form a giant cell (i.e., zygote). This giant cell ingests other amoebae that stream in, responding to the same chemoattractant used in the social stage, and increases in size [18,22]. Then, the macrocyst forms a cellulose wall, before meiosis and cleavage occur, and eventually releases hundreds of haploid amoebae. This sexual stage has been reported in several dictyostelid species [18,22,24,25], including some species with strains that are self-compatible (i.e., homothallic) as well as cross-compatible (i.e., heterothallic), such as the most well-known and best-studied dictyostelid, *D. discoideum*. One to six mating types have been reported in different species [e.g., [24,26,27]].

Morphological characters associated with both asexual fruiting body formation and sexual macrocyst formation have been traditionally used to classify the dictyostelids [28]. However, recent work by Schaap et al. [29] reconstructed a phylogeny of the Dictyostelia using DNA sequence data from multiple loci and found extensive genetic variation among dictyostelid species. Mehdiabadi et al. [27] examined within species variation in greater detail for the social amoeba *Dictyostelium purpureum *and showed strong intraspecific genetic differentiation - some haplotypes found within *D. purpureum *were more divergent than a number of pairs of closely related but distinct species, suggesting the possibility of cryptic species. The objectives of the current study are (1) to examine the evolutionary history of *Dictyostelium giganteum *by sequencing the same regions of the nuclear ribosomal DNA, and using this phylogeny (2) to compare the level of intraspecific genetic variation between *D. giganteum *and *D. purpureum *and (3) to test predictions on the potential for sexual mating (macrocyst formation) between clones of *D. giganteum *with varying genetic and/or geographical distances. This work is fundamental to understanding social behavior among clones of *D. giganteum *[e.g., see [30]], a dictyostelid with a wide geographic distribution [31], because interactions between species have very different evolutionary trajectories than social interactions within species.

## Results

### rDNA gene tree

We sequenced, on average, 4,060-bp of the rDNA in all 24 samples and identified 16 unique haplotypes. With the two published *D. giganteum *species (GenBank accession numbers AF219102 and AM168042[29]) this makes 18 unique haplotypes for use in the analyses. The most common haplotype (haplotype 15) was present in four isolates (Additional file 1). For all unique ribosomal DNA haplotypes (including outgroups), we aligned 4,309 sites (which includes gaps and insertions) of which 509 were variable and 326 were parsimony informative [[32]; Additional file 1, Figure 2]. However, if we restrict our analysis to only *D. giganteum *haplotypes, we found 26 variable sites, (45 variable sites if polymorphic indels are included), of which 16 were parsimony informative.

**Figure 2:**
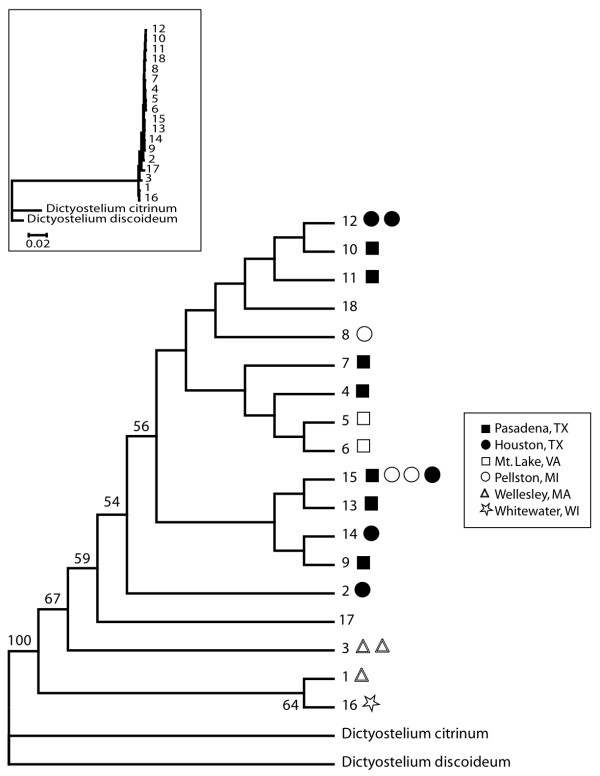
**Bayesian phylogeny of 18 unique haplotypes of *D. giganteum***. Isolates QSgi11, QSgi14, and QSgi15 are not shown due to uncertainty on their haplotype assignment, but all could belong to either haplotype number 14 or 15. QSgi15 could also belong to haplotype 12. Published sequences of *D. citrinum *and *D. discoideum *served as outgroups. Nodes with posterior probabilities below 50% are not shown. Symbols refer to geographical locations of isolates. Inset shows genetic distances between haplotypes.

Neighbor-joining, maximum parsimony (MP), maximum likelihood (ML), and Bayesian analyses of the unique haplotypes produced similar topologies. Figure 2 shows the rDNA gene tree using only the Bayesian approach. All analyses revealed weak evidence of phylogenetic structure despite the Massachusetts and Wisconsin isolates coming out at the base of the trees and the two Mt. Lake, VA haplotypes being genetically distinct but sister nodes in all trees except for the MP tree. Regardless, analyses of sequence data found very little genetic differentiation among isolates, and overall, relationships within *D. giganteum *were not well resolved in any of the trees.

We found small genetic distances between groups of lineages, substantially smaller than differences between closely related but distinct species. The average genetic distance between *D. giganteum *and outgroup taxa was 0.110 (range 0.103 - 0.111), and 0.037 between outgroups *D. discoideum *and *D. citrinum*. Within *D. giganteum*, genetic distances ranged from 0 - 0.022 (including the two published *D. giganteum *sequences) and 0 - 0.007 (excluding the two published *D. giganteum *sequences). The average pairwise sequence divergence between the basal lineages (i.e., the Massachusetts and Wisconsin isolates) and the rest of the *D. giganteum *clones was 0.004.

Overall, we found pronounced population differentiation for *D. giganteum *(Fst = 0.67, P < 0.0001). Pairwise population comparisons indicated that this was largely driven by the Massachusetts group being different from both Texas populations (Pasadena, TX vs. Wellesley, MA: F_ST_: 0.816, p = 0.0039; Houston, TX vs. Wellesley, MA: F_ST_: 0.738, p = 0.0127) given that no other pairwise population comparisons were significant (data not shown). Nevertheless, the sample sizes are very small, so power to test further structure is lacking.

### Mating Experiments

The findings of low genetic divergence within *D. giganteum*, compared to the divergence between species, suggests that *D. giganteum *is a single species and sexual mating (i.e., macrocyst formation) in *D. giganteum *is just as likely to occur between pairs of isolates from throughout the tree and/or different geographic locations, assuming isolates are of different mating types. The two groups we focused on were the group consisting of the basal lineages (i.e., the Massachusetts and Wisconsin isolates) and the group containing the rest of the *D. giganteum *isolates given that F_st _estimates showed significant differentiation between the Massachusetts and Texas populations.

**Figure 3:**
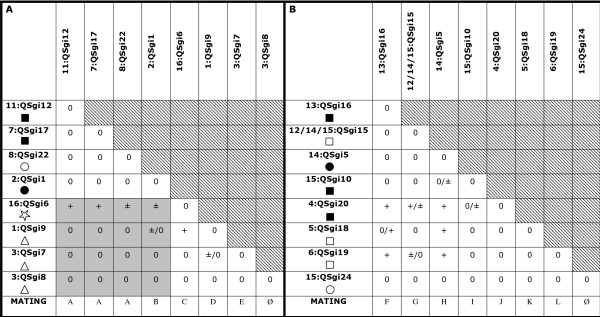
**Results of Macrocyst Experiments #1 (panel A) and #3 (panel B)**. Each of the three non-overlapping experiments consisted of all possible pairwise matings between 8 isolates (isolate names are given as haplotype number followed by isolate number, and symbols under isolate names refer to their geographical location; see Figure 2 legend). A "+"indicates that macrocysts formed between a pair of isolates within one week of scoring, a "±" indicates that macrocysts formed after the standard one week of scoring, and a "0" designates that macrocysts did not form between a given pair of isolates. Specific pairwise matings were replicated for the two pairs with two symbols in Experiment #1 (panel A) and all pairs of Experiment #3 (panel B). Cases where first and second replicates did not give similar results are designated with two symbols in a given cell. Grey-colored cells represent pairwise matings between isolates from the basal lineages and other clones given that F_st _estimates showed significant differentiation between the Massachusetts and Texas populations. Mating types are shown in the last row: different letters indicate different mating types, and "∅" represents clones that did not mate with any other clones. (Macrocyst Experiment #2 did not produce any successful matings and thus, results are only mentioned in the text.)

To test the above hypothesis, we performed three sets of 8-clone pairwise mating experiments with isolates that varied in both geographical and genetic distances. After replicating all of Experiment #3 and two other pairwise matings from Experiment #1 (see Figure 3), we found our experimental design produced results that were repeatable 95% of the time (i.e., 36/38 matings gave similar results after the standard one week of scoring). However so few of our clones formed macrocysts under any circumstances that it is likely that our conditions were not optimal for macrocyst production.

In all three experiments, we found that no isolates were homothallic (no self mating), which confirmed previously published results for this species [26]. Ten of the 24 isolates did not form macrocysts in any combination (Figure 3), including all eight isolates in Experiment #2: QSgi14 (haplotype number 14/15), QSgi21 (haplotype number 10), QSgi13 (haplotype number 9), QSgi4 (haplotype number 12), QSgi23 (haplotype number 15), QSgi3 (haplotype number 12), QSgi11 (haplotype number 14/15), and QSgi2 (haplotype number 15). In addition, one clone, QSgi10, produced only one to a few macrocysts with only two other isolates (Figure 3b). In each of the three 8-clone experiments, we had a total of 7/36, 0/36, and 10/36 macrocysts form, respectively (Figure 3). This resulted in at least 7 apparent mating types or sexes (Figure 3). We defined mating types to be exclusive with no overlap.

For example, in Experiment #1, QSgi12, QSgi17, and QSgi22 were all considered the same mating type because all mated with QSgi6 but no other isolates did (Figure 3a). Overall, the data confirmed our hypothesis: there was no significant difference in the number of successful within and between group matings regardless of the time at which macrocyst formation was scored, neither after one week of scoring (within: 8/92, between: 2/16; Fisher's exact test, p = 0.6410), nor after four weeks of scoring (within: 12/92, between: 5/16; Fisher's exact test, p = 0.1278).

We also investigated whether the time to macrocyst formation for these pairs correlated with genetic distance between a pair of clones but found no significant relationship (Non-parametric Spearman Rank Correlation: Z = 1.265, p = 0.206). The same was true for geographical distance (Non-parametric Spearman Rank Correlation: Z = -1.505, p = 0.132).

## Discussion

Several phylogeographic inferences can be made about *D. giganteum *from the phylogenetic analyses of the rDNA sequence data. First, and most importantly, there appears to be very little genetic differentiation among isolates of *D. giganteum *and no clear evidence of phylogenetic structure. Isolates from a given geographical location do not cluster together (Figure 2). The Wisconsin and Massachusetts isolates are basal on the Bayesian (Figure 2), ML, MP, and neighbor-joining trees, indicating that isolates sampled from the north tend to be basal to isolates sampled from the south (with the exception of the Michigan clones).

Our results are very different than those found in a previous study for another dictyostelid species *D. purpureum *[27]. There is considerably more genetic variation and phylogenetic structure in *D. purpureum *than *D. giganteum*. Mehdiabadi et al. [27] also sequenced the same regions of the nuclear ribosomal DNA that we did for *D. giganteum*, yet found pairwise genetic distances between some *D. purpureum *haplotpes to be more than twice as divergent than pairwise distances between taxa that are recognized as closely-related but distinct species. Similar results were also found for the two *D. purpureum *clones used in Schaap et al. [29]. Isolates sampled for *D. giganteum *in the present study were more geographically dispersed throughout the United States but less genetically variable than the isolates sampled for *D. purpureum *in Mehdiabadi et al. [27]. What accounts for these differences between the two dictyostelid species remains unknown. However, we also cannot rule out the possibility of a higher level of genetic variation for *D. giganteum *than observed if we included isolates from a wider geographic distribution.

Since there was no clear evidence of phylogenetic structure and there was a low level of genetic differentiation in the rDNA across haplotypes for this species, we predicted that sexual mating should occur between a pair of *D. giganteum *isolates across the species (given they were different mating types) regardless of geographical location or genetic distance. This is in contrast to *D. purpureum *- a species with extensive genetic variation and phylogenetic structure. Mehdiabadi et al. [27] predicted and found reduced sexual compatibility between *D. purpureum *isolates from different phylogenetic groups. However for *D. giganteum*, we found that sexual mating (macrocyst formation) did not correlate with either genetic or geographical distance (Figure 3). For example, QSgi6 (collected in Wisconsin) and QSgi9 (collected in Massachusetts) formed macrocysts despite differences in geography (Figure 3). Furthermore, these two isolates were just as likely to mate with isolates separated by relatively large genetic distances as they were with isolates with very small genetic distances (Figure 3). We found for some pairs that if after the first week macrocysts did not form, that over time, they would eventually produce macrocysts (see Figure 3).

Although our predictions were confirmed that there was no correlation with either genetic or geographical distance and macrocyst formation, we did find a few inconsistent and ambiguous results, as previous work has shown in similar studies of macrocyst formation in *Dictyostelium*. For example, experiments by Erdos et al. [26] also found that several strains of *D. giganteum *did not form macrocysts with any other strain they were paired with, and this happened for ten of our 24 isolates. In addition, they also found inconsistent mating patterns between some pairs of strains [26]. Based on our findings, inconsistent mating reactions could be attributable to the time at which macrocyst formation is scored. That is, matings might be more likely to occur between a pair of clones, the more time the pair is given to mate. Scenarios like this are known in other systems. For example, in fiddler crabs, females are less choosy in their male partners as search time for males increases [33], and similar results have been found in bushcrickets [34]. In our experiments, a given clone had only one potential partner available for mating. Another possible explanation for inconsistent matings between pairs of isolates is that pheromones, which have been found to induce macrocyst formation in *D. giganteum *strains [35], may not be produced (or may even be inhibited) under certain circumstances. Thus, we also cannot rule out the possibility of imperfect environmental conditions for all possible pairwise matings as a reason for inconsistent mating reactions.

## Conclusions

Clearly, the North American isolates of *D. giganteum *comprise a single species, which means that social theory is applicable to interactions among clones. Genetic distance between interacting pairs may influence the nature of their interactions. In *Dictyostelium*, both Mehdiabadi et al [27] and Ostrowski et al. [36] found a positive correlation in the degree of mixing between a pair of isolates and their genetic distance for *D. purpureum *and *D. discoideum*, respectively. In a related study, Kaushik et al. [30] conducted pairwise mixtures among five isolates of *D. giganteum *from India and found that when different clones mix, they form predominantly clonal fruiting bodies and only sometimes form chimeric fruiting bodies. These differences in the degree of chimeric fruiting body formation between pairs of clones may be due to their sequence divergence. Future work is needed to determine whether the degree of mixing correlates with genetic distance for this species as has been found for other dictyostelid species [27,36] and to understand what accounts for such different patterns of intraspecific genetic variation of this important group of eukaryotic social microbes.

## Methods

### Samples

We used 24 *D. giganteum *isolates, which were collected from 6 different geographic locations around the United States: (i) 9 clones from Pasadena, Texas (29° 35' N, 95° 4' W), (ii) 5 clones from Houston, Texas (29° 46' N, 95° 27' W), (iii) 3 clones from the University of Michigan Biological Station near Pellston, Michigan (45° 33' N, 84° 40' W), (iv) 3 clones from Wellesley, Massachusetts (42° 17' N, 71° 18' W), (v) 3 clones from Mountain Lake, Virginia (37° 21' N, 80° 31' W), and (vi) 1 clone generously provided by Jim Cavender from Whitewater, Wisconsin (Additional file 1). Isolates were frozen as pure cultures (i.e., development of fruiting bodies arising from a single spore) for permanent storage. We also included published sequences of two *D. giganteum *samples, one collected from Wisconsin (isolate WS589) and another from an unknown location (GenBank accession numbers AM168042 and AF219102, respectively; Additional file 1). Samples of *Dictyostelium discoideum *(GenBank accession number X00601) and *Dictyostelium citrinum *(GenBank accession number DQ340385) served as outgroups.

### Molecular work and data analyses

DNA extraction, amplification, and sequencing were carried out as described in Mehdiabadi et al. [27]. We extracted DNA from clones by placing 5-10 individual sori (the cluster of spores at the top of the fruiting body) in 150 μL of 5% Bio-Rad Chelex and 10 μL of proteinase K and ran the samples in a PTC-100 programmable thermal controller (step 1: 56.0°C for 4 h; step 2: 98.0°C for 30 min). From each of the 24 isolates, we amplified regions of the nuclear ribosomal DNA (one locus; ~4,000-bp total; [see [27]]) in 10 μL polymerase chain reactions (PCR; 1.125 μL MgCl_2_, 0.2 μL DNTPs, 1 μL forward primer, 1 μL reverse primer, 1 μL 10× Buffer, 0.1 μL Platinum Taq DNA Polymerase (Invitrogen), 4.575 μL water, and 1 μL DNA) using the following protocol (step1: 94.0°C for 2 min; step2: 94.0°C for 30 sec; step 3: 65.0°C decreasing 1.0°C every 30 sec cycle; step 4: 72.0°C for 1 min; step 5: 15 cycles to step 2; step 6: 94.0°C for 30 sec; step 7: 50.0°C for 30 sec; step 8: 72.0°C for 1 min; step 9: 25 times to step 6; step 10: 72.0°C for 15 min), sequenced PCR products in both directions, and performed phylogenetic analyses. We selected the nuclear ribosomal DNA as the marker of choice because Schaap et al. [29] showed that it can resolve differences between closely-related dictyostelid species. Furthermore, this molecular marker has been used in previous studies assessing intraspecific genetic variation in other dictyostelid species and has proven to be suitable for such work [27]. We assembled contigs for individual clones in SeqMan (Lasergene version 7.0; DNASTAR, Inc., Madison, WI) and aligned sequences using ClustalW [37] in BioEdit version 7.0.0 [38]. Sequences have been deposited in GenBank under accession numbers GU386290-GU386313.

We used four different methods to reconstruct the rDNA gene tree: Bayesian, ML, MP, and neighbor-joining approaches. For the Bayesian tree, we used MrBayes v3.1 [39] to estimate a phylogeny of the unique haplotypes based on the GTR+I+G model of molecular evolution. Four Metropolis-coupled Markov chains were run for 250,000 burn-in generations followed by 1.75 × 106 generations of data collection. We used GARLI [40] to infer the ML bootstrap tree with 1000 bootstrap pseudoreplicates under the GTR+G model, which was selected by the Akaike Information Criterion in ModelTest v. 3.06 [41]. MP analyses were conducted in PAUP*v.4.0b10 [42] with 1000 bootstrap replicates using tree bisection-reconnection (TBR) branch swapping and 10 random-taxon-addition replicates per bootstrap pseudoreplicate. The neighbor-joining gene tree of unique haplotypes was reconstructed using MEGA4 [32]. Bootstrap values were based on 1000 replicates.

We estimated genetic distances between haplotypes in MEGA4 [32] using the p-distance model. All results were based on the pairwise analysis of 28 sequences, and all positions containing alignment gaps and missing data were eliminated in pairwise sequence comparisons (pairwise deletion option).

To test for population differentiation, we calculated F_ST _with the rDNA sequence data using the analysis of molecular variance approach [43] implemented in Arlequin 2.0 [44]. Our analysis is based on 2,410 bases (all positions with less than 5% missing data).

### Mating experiments

To test whether mating correlated with genetic distance, we performed three round robin 8-clone macrocyst experiments, where each clone was paired with seven other clones as well as itself (i.e., to determine whether any clones were self-compatible; Figure 3). This resulted in a total of 36 pairings for each experiment. Thus, 108 potential matings were carried out. In addition, we repeated macrocyst experiments for all pairings in Experiment #3 as well as two pairings from Experiment #1 to confirm the repeatability of our results (Figure 3). We assigned clones to experiments based on their position in the gene tree as well as to their geographical location. Experiments were performed as described in Mehdiabadi et al. [27]. To infer successful matings, we recorded the presence or absence of macrocysts for each pair over time for the entire duration of the experiment: after one week, two weeks, three weeks, and four weeks. However, viability of progeny was not determined.

### Data analyses for mating experiments

To analyze the results of the macrocyst experiments, we used a Fisher's exact test to statistically determine whether mating was random or whether it correlated with geographical and/or genetic distance. We also tested whether the time to macrocyst formation correlated with either genetic or geographical distance of a given pair of clones by using a nonparametric Spearman Rank correlation. For the few cases where one replicate resulted in macrocyst formation and the other replicate did not (after one week of scoring: 1/38; after two or more weeks of scoring: 6/38), we considered that pair as capable of forming macrocysts. Also, if replicate experiments for a given pair of clones had macrocysts form at different times (1/38), we used the earliest time in our Fisher's exact test analysis and the averaged time in our correlation analysis.

## Authors' contributions

NJM, MRK, DCQ, and JES designed research, NJM performed research, NJM and MRK analyzed the data, NJM, MRK, DCQ, and JES wrote the manuscript, and all authors read and approved the final manuscript.

## Supplementary Material

Additional file 1 **Table S1**. D. giganteum unique haplotypes. Symbols refer to geographical locations of isolates as shown in Figure 2. Click here for file
