# Supplementary material for: Phylogeography and sexual macrocyst formation in the social amoeba Dictyostelium giganteum
Source: BMC Evol Biol. 2010 Jan 20;10:17. doi: 10.1186/1471-2148-10-17 (PMC2824659; doi:10.1186/1471-2148-10-17)
Supplement: Additional file 1 — Table S1. D. giganteum unique haplotypes. Symbols refer to geographical locations of isolates as shown in Figure 2. [file 1471-2148-10-17-S1.PDF]

Additional file

Table S1 - *D. giganteum* unique haplotypes. Symbols refer to geographical locations of isolates as shown in Figure 2.

Haplotype Number	Isolates Belonging to that Haplotype
1	QSgi9 △
2	QSgi1 ●
3	QSgi7 △, QSgi8 △
4	QSgi20 ■
5	QSgi18 □
6	QSgi19 □
7	QSgi17 ■
8	QSgi22 ○
9	QSgi13 ■
10	QSgi21 ■
11	QSgi12 ■
12	QSgi4 ●, QSgi3 ●(possibly QSgi15 □)
13	QSgi16 ■
14	QSgi5 ● (possibly QSgi15 □, QSgi11 ■, QSgi14 ■)
15	QSgi10 ■, QSgi24 ○, QSgi23 ○, QSgi2 ● (possibly QSgi15 □, QSgi11 ■, QSgi14 ■)
16	QSgi6 ☆
17	D. giganteum reference sequence (GenBank accession number: AM168042)
18	D. giganteum reference sequence (GenBank accession number: AF219102)
